# Strengthening Health Systems for Persons With Traumatic Spinal Cord Injury in South Africa and Sweden: A Protocol for a Longitudinal Study of Processes and Outcomes

**DOI:** 10.3389/fneur.2018.00453

**Published:** 2018-06-14

**Authors:** David Conradsson, Anthea Rhoda, Nondwe Mlenzana, Lena Nilsson Wikmar, Kerstin Wahman, Claes Hultling, Conran Joseph

**Affiliations:** ^1^Function Area Occupational Therapy and Physiotherapy, Allied Health Professionals Function, Karolinska University Hospital, Stockholm, Sweden; ^2^Physiotherapy Division, Department of Neurobiology, Care Sciences and Society, Karolinska Institutet, Stockholm, Sweden; ^3^Physiotherapy Department, Faculty of Community and Health Sciences, University of the Western Cape, Cape Town, South Africa; ^4^Section Neurorehabilitation, Division of Neurodegeneration, Department of Neurobiology, Care Sciences and Society, Karolinska Institutet, Solna, Sweden; ^5^Spinalis Research and Development Unit, Rehab Station Stockholm, Stockholm, Sweden; ^6^Spinalis Foundation, Stockholm, Sweden

**Keywords:** traumatic spinal cord injury, specialized care, health systems, acute care, outcomes, South Africa, Sweden

## Abstract

**Background:** The provision of specialized care in a time-sensitive manner has shown to be crucial for survival and recovery of functioning after a traumatic spinal cord injury (TSCI). However, little is known about the provision of TSCI care in different international contexts; information which is required for strengthening policy and practice.

**Aims:** The overarching aim of this study will be to explore health care processes and outcomes of TSCI care in South Africa and Sweden. Specific aims will be to: (1) describe acute processes of TSCI care, (2) determine acute- and long-term outcomes of TSCI care, and (3) identify predictors for survival, secondary complications, and functioning 12 months post-injury.

**Methods:** A prospective (regional), population-based cohort study where adults with an acute TSCI will be recruited over at least a 1-year period from the City of Cape Town, South Africa, and Stockholm, Sweden. The anticipated sample size inclusive of both international contexts will be 200 participants—based on a power calculation for detecting differences in mortality. Information on the nature and timing of processes of acute care (e.g., transfer logistics, spinal surgery, and specialized SCI care) will be collected on acute care admission and discharge using a standardized form. Survival status, secondary complications, neurological symptoms, functional status, activity, and participation as well as health-related quality of life will be collected at discharge from SCI acute care and at 12-months post-injury. Secondary complications and functioning will be compared between South Africa and Sweden using inferential statistics. To address mortality specifically, the indirect standardization method for differences in mortality between contexts will be used whereby Stockholm will serve as standard for specialize care. For the assessment of factors related to mortality and other outcomes (e.g., neurological and secondary health conditions) multivariate regression analyses will be used to determine independent risk factors.

**Conclusion:** This study offers a unique investigation of the relationship between health care processes and outcomes of TSCI care with the aim of strengthening management guidelines for SCI in South Africa and Sweden.

## Introduction

A traumatic spinal cord injury (TSCI) often causes an unprecedented change in functioning by altering bodily structure and function (1). More specifically, the direct consequences of TSCI to the motor, sensory and autonomic nervous system not only challenge an individual's independency but also the ability to make a positive adjustment to life after injury (2, 3). In line with this, TSCI survivors often experience threats to their livelihood and becoming integrated members of society (4, 5). Health systems therefore need to be ready to respond to the myriad of challenges following a TSCI by providing access to specialized and comprehensive services (6).

Like many unforeseen medical emergencies, a TSCI could lead to early death in the absence of essential emergency and acute care (7, 8). Hence, the single most important indicator of the quality of medical care is reflected in short- and long-term mortality (9). However, in order to improve survival after TSCI, it is also important to identify factors and processes of routine practice related to mortality. This could be accomplished by examining the extent to which processes of care reflect international guidelines and standards (10, 11). These guidelines specifically stress the importance of a systematic approach toward the management of TSCI, which include specialized and comprehensive care that is delivered in a multi-disciplinary manner. More specifically, established success factors of TSCI healthcare have proven to include appropriate emergency management (e.g., transfer logistics to trauma unit) (12, 13), early spinal surgery (6, 14) and admission to specialist units (15–17), as well as multidisciplinary care (17). As a collective, literature has shown that a systematic approach toward the management of TSCI leads to a reduction in mortality, secondary complications and re-hospitalizations, as well as better functional outcomes (16–18).

In Sweden, a systematic approach toward the management of TSCI was initiated around the 1970's (19). A number of studies investigating the acute management and mortality after TSCI found better aligned processes of care, and significantly greater survival, in Sweden, compared with a non-systematic approach to care followed in Greece (15, 16, 18). More specifically, transfer logistics from the accident scene to a specialized trauma unit and key therapeutic interventions better reflected clinical guidelines in Sweden. Furthermore, Divanoglou et al. demonstrated for the first time that key interventions, such as timing of spinal surgery, are related to better clinical outcomes, for example survival and neurological recovery in Sweden (15, 16, 18). In addition, numerous societal services, such as vocational rehabilitation, modifications of living conditions and workplace insurance, are provided to survivors of TSCI to enhance their independence and societal participation (20).

Unlike Sweden, South Africa had yet to fully implement a systematic healthcare approach to TSCI management. Access to specialized healthcare services is not only a historic problem in South Africa, but it is further exacerbated by the lack of resources. Only one specialized SCI unit is available in South Africa, serving around 8 million people in the Western Cape Province. With the high incidence of TSCI in South Africa (21), mainly high risk TSCI patients (e.g., those in need of stabilizing spinal surgery) get access to specialized care at the SCI unit (22). In contrast, those not prioritized for treatment at the specialized SCI unit are treated at hospitals, including both secondary and tertiary level of care, providing non-specialized SCI health care. The common occurrence of non-specialized care of TSCI survivors in South Africa is alarming, especially as such care has shown to be related to a high mortality rate and the occurrence of preventable secondary complications (23, 24). To improve the outcome of TSCI in South Africa, there is a crucial need to audit the nature and timing of essential processes of care and model their relationship with mortality, complications, and long-term outcomes. Furthermore, given the low 1-year mortality in Sweden (16), a comparison of processes of care between Sweden and South Africa could provide empirical evidence pointing to processes which could be modified.

As a first step to strengthening the management guidelines for TSCI care in South Africa, the overarching aim of this study is to explore health-care processes and outcomes of TSCI care in South Africa and Sweden. Specific aims are to: (1) describe acute processes of TSCI care in South Africa and Sweden in comparison to international guidelines, (2) determine acute- and long-term outcomes of TSCI care, including survival status, secondary complications and functioning in South Africa and Sweden, and (3) identify predictors for survival, secondary complications and functioning 12 months post-injury in South Africa and Sweden.

We hypothesize that processes of care following SCI will be better aligned with international guidelines and standards in Sweden but not in South Africa. We further expect worse acute- and long-term outcomes, e.g., survival, neurological recovery, and health-related quality of life, for survivors of TSCI in South Africa compared to Sweden due to differences in the health-care systems.

## Methods

### Design

**Figure 1:**
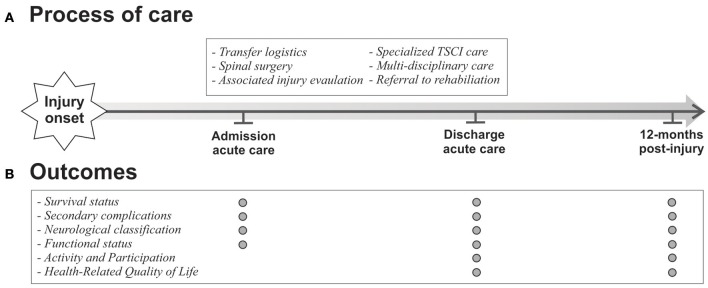
Overview of processes and outcomes of care.

To this prospective, regional population-based cohort study we will recruit all adults with an acute traumatic SCI diagnosis in the Metropolitan area of Cape Town, South Africa and the greater Stockholm area, Sweden, over at least a 1-year period. As illustrated in Figure 1, data will be collected at admission to and discharge from SCI units and 12 months post-injury. Due to the descriptive and analytical nature of study aims 1 and 2, selected pre-hospital processes of care information and baseline measurement of outcomes will be collected within 7 days post-injury. This study constitutes an observational design where all those identified in year 1 will be followed for 12 months for the evaluation of mortality and functioning (see Figure 1B). In line with the assumptions of causality, this study design provides favorable conditions for modeling indicators of mortality in both the short- and long-term.

### Settings

South Africa is classified as a low-to-middle income country where vast discrepancies exist in socio-economic standing and healthcare provision and access (25). The City of Cape Town Metropolitan area is home to 3.74 million persons, spread across 2,445 km^2^, consisting of both urban and peri-urban areas. The catchment area has two level 1 hospitals which has the organizational capacity to manage SCI, still only one of them provides specialized SCI care. The SCI unit has six intensive care unit beds, and because of this selection of patients are based on priority which consequently lead to many survivors of TSCI being acutely managed in general units across numerous secondary hospitals.

Sweden, as a high-income country, will be used as criterion, since they have developed a systems approach toward SCI management which has a low to non-existing 12-months mortality rate and a high return-to-work rate after 2 year (15, 16). The population size of the greater Stockholm area at the end of 2016 was estimated to be 2. 27 million. Within this region, several level 1 trauma units are available which provides immediate and comprehensive intensive care, one spinal cord injury unit providing post-acute/primary rehabilitation, three active rehabilitation units and one outpatient clinic delivering life-long follow-up care.

### Population, inclusion criteria, and sample size

In line with recent studies, we will include patients with a TSCI after 7 days post-injury (21, 26) during the surveillance year fulfilling the following inclusion criteria:

- A TSCI defined as a sudden loss of voluntary muscle strength, sensation and autonomic functions below the level of injury, which will vary depending on neurological level of injury and extent of impairment, but must include altered sacral sensation (27);
- The injury must result in persisting impairment (i.e., not just a concussion) after emergence from neurogenic shock, which generally occurs within the first 24–72 h after injury;
- Abnormal imaging, such as with Magnetic Resonance Imaging scan or multi-slice Computed Tomography scan;
- ≥18 years of age;
- Residents of one of the two study settings;
- Those admitted to the government-funded hospitals providing SCI care;
- Those consenting to participate in the study

In this study, 1-year mortality rate will be used as primary outcome. A power calculation, at 80% power and Beta 0.2, was performed to determine the number of participants to be included per country, assuming a mortality rate of 35% in South Africa and 5% in Sweden. Detecting a 30% difference in mortality would require the enrollment of 50 persons per country. Assuming standard approximations for loss to follow up, we expect that 55 persons in each international context would be sufficient to retain statistical power. We will, however, continue recruitment for at least a 1-year cycle in order to capture seasonal variation in incidence and mortality which may increase the total sample to approximately 200 persons. By continuing the study period for 1 year we will further be able to provide a more heterogeneous study population. Previous incidence studies in Cape Town (21) and Stockholm (28) suggest the attainment of these recruitment targets.

## Data collection

This study will be carried out in accordance with the recommendations of Guidelines for Good Clinical Practice. The protocol was approved by the University of the Western Cape's Biomedical Science Research Ethics Committee [Ref number: BM 16/3/24], and all subjects will be required to provide written informed consent in accordance with the Declaration of Helsinki. This prospective, observational, cohort study was registered with ClinicalTrials.gov [NCT03437850]. Data will be collected at three time points; (1) All participants, in both contexts, will be evaluated on admission to the acute SCI units within 7 days of admission. One experienced physiotherapist in both settings will be responsible for screening every consecutive patient with acute TSCI for eligibility in this study. Thereafter, all eligible patients will be asked for informed consent following a detailed description of the study. This is also the time point where pre-hospital and emergency care data will be collected, such as time to level 1 trauma unit, any intermediate hospital admissions, time to imaging, and assessment and time to planned surgery, as well as all outcomes. (2) The remaining selected processes of care and outcomes will be measured at the end of acute care. Thereafter, outcomes will be measured at (3) 12 months post-injury.

### Data collection instruments

This section outlines data collection with respect to instruments that will be used to (1) describe the two cohorts, (2) processes of care, and (3) outcomes during the acute care as well as 1-year follow up. Importantly, the core sets for SCI will be used as functioning indications, where psychometrically sound measures which are aligned with these indicators will be used as outcomes. All outcome measures chosen have been recommended by the International Spinal Cord Society.

## Instruments describing participants' characteristics

The *International Spinal Cord Injury Core Data Set* will be used to collect information about age, gender, etiology, whether spinal surgery was performed, the presence of significant associated injuries (e.g., bony vertebral injury), neurological classification according to international standards, length of hospital stay during acute care, and place of discharge (i.e., home, inpatient rehabilitation or out-patient rehabilitation) from acute care (27, 29).

## Processes of care

Processes of acute care were selected based on the strongest level of evidence indicating their influencing on mortality and functioning. These selected processes have been included as part of acute clinical practice guidelines for managing persons with SCI (6, 30). Table 1 outlines important care processes which will be consistently collected in both study contexts, as well as how these will be measured and captured. The following secondary medical complications will be screened for, as contained in the Secondary Complications Scale—Spinal Cord Injury: pressure ulcers, injury caused by loss of sensation, muscle spasms, contractures, heterotopic bone ossification, diabetes mellitus, bladder dysfunction, bowel dysfunction, urinary tract infection, sexual dysfunction, autonomic dysreflexia, postural hypotension, circulatory problems, respiratory problems, and joint, and muscle pain. See Supplementary Material 2 for operational definitions of all secondary complications.

**Table 1:** Acute TSCI care processes.

**Acute care processes**	**Outcomes**
Transfer logistics	Time (minutes) from accident scene to level 1 trauma unit
Spinal surgery	Decompression/stabilizing spinal surgery (Yes/No) Time (hours) to receive decompression/stabilizing spinal surgery
Associated injuries evaluation	Screening for associated injuries (Yes/No) Use of golden standard diagnostic tools, e.g., MRI/CT
Specialized SCI care	Time (days) from accident scene to specialized SCI unit admission Number of intermediate hospitalisations
Multi-disciplinary team	Was the patient managed by a multi-disciplinary team, i.e., defined as a medical doctor, nurse, rehabilitation personnel (Yes/No)
Secondary complications prevention	Screening for secondary complications (Yes/No) What type of screenings tests were used? What type of secondary complications were screened for?
Length of acute care	Time (days) from first hospitalization until the end of acute care (including all intermediate care)
Rehabilitation	Referral to inpatient or outpatient rehabilitation (Yes/No)

### Outcomes after TSCI according to the international classification of functioning, disability, and health

The *International Classification of Functioning, Disability and Health (ICF)* conceptualizes health and disease/illness in terms of impairments, activity limitations and participation restrictions. SCI-specific comprehensive and brief core sets containing relevant and prototypical functioning categories for SCI have been developed, which will be used as functioning indicators (31, 32). These core sets will be used to assess the impact of healthcare services on the attainment of disease-specific outcomes, but also for identifying unmet needs following different healthcare episodes.

Outcomes were selected in order to reflect the influence of SCI on the entire spectrum of functioning, as conceptualized within the ICF. Since the ICF is an exhaustive framework, the core sets were used to select appropriate outcome measures according to the ICF domains (31, 32). Table 2 outlines the outcome measures that will be used, and Supplementary Material 1 contains the categories of functioning targeted by each outcome measure to illustrate the coverage of relevant information for SCI.

**Table 2:** Outcomes of care.

**Outcomes**	**Indicator/Measure**	**Validity and reliability**
**HEALTH OUTCOMES**		
Survival status	Alive or dead	Death certificate
Secondary complications	Secondary health conditions scale-SCI	Internal consistency; test-retest reliability; convergent validity (33)
**FUNCTIONING OUTCOMES**		
Neurological classification	International Standards of assessment (ASIA)	Internal consistency; test-retest reliability; construct validity (27, 34, 35)
Functional status	SCIM III	Inter- and intra-rater reliability; construct validity (36–38)
Activities/participation and return to work	CHART European Quality of Life	Inter-and intra-reliability; construct validity (39)
Health-related quality of life	5D Qol ISCoS Data Set	Valid and reliable measure of utility (40)

*ASIA, American Spinal Injury Association; SCIM III, Spinal Cord Independence Measure version III; CHART, Craig Hospital Assessment and Reporting Technique*.

### Data analyses

Data will be captured and stored in an encrypted Microsoft Excel file. Following data collection, random data entry checks will be conducted to ascertain its accuracy. Data analysis will be carried out in relation to the study objectives, with processes of care being analyzed descriptively. To address the primary outcome—mortality—specifically, the indirect standardization method for differences in mortality between contexts will be used whereby Stockholm will serve as standard for international care. Secondary outcomes, for example AIS grade conversion, functional recovery, and prevalence of secondary medical conditions will be compared between South Africa and Sweden using inferential statistics, depending on the normality of data. For the assessment of factors related to mortality, predictors will be clustered into several categories reflecting the hierarchical structure as proposed by Krause's theoretical risk model for mortality and secondary complications: (1) injury-related factors (i.e., cause of injury, level and severity of injury, vertebral fractures, ventilatory support, and associated injuries); (2) socio-demographic factors (i.e., sex, age, socio-economic position, ethnicity, rurality); and (3) processes of care/environmental factors (e.g., number of intermediate hospitalization, time to surgery, multi-disciplinary team managing patient).

For determining the risk associated with mortality, potential risk factors, according to the categories above, will be added one at a time in unadjusted univariate logistic regression models. At this point, significant predictors will be set at alpha level equal to 0.10 for further inclusion in the multivariable analyses. Lastly, predictors will be entered into a multivariable logistic regression model where confounders will be added if causing a chance in β which exceeds 10% (unstandardised regression coefficient) of the independent variables. At this stage, the statistical significance level will be set at *P* ≤ 0.05.

## Discussion

This proposed study will closely investigate key processes of acute care that are likely to influence survival rates and recovery in terms of impairments, activity, participation, and quality of life following TSCI. Information derived from this study could be used to strengthen systems of care for individuals with TSCI in South Africa and Sweden.

A significant gap of knowledge regarding the nature of TSCI care continues to exist in low-to-middle income countries. This project was conceived in the aftermath of the International Perspectives on Spinal Cord Injury (41) which highlighted the current successes of specialized approaches in managing SCI in Western countries. This report further highlighted the dire living conditions and lack of available resources for persons with SCI in developing countries by specifically noting the lack of research underpinning essential epidemiological data as well as identifying the unmet needs of survivors due to the lack of monitoring and evaluation of mortality and health post-injury (42). Therefore, by contrasting non-specialized care in South Africa and specialized TSCI care in Sweden this study provides a unique opportunity to develop and strengthen clinical practice guidelines for TSCI care in South Africa. The present study will further expand beyond the outcome of TSCI care by investigating whether acute care events and processes could predict mortality and functioning states 12 months post-injury.

Success of health care is often assessed in terms of mortality, an outcome easily captured in routine health care practice. In order to address this pivotal outcome it remains necessary to apply models which could help assist the identification of factors pertinent to the survival of individuals. The scope of processes and outcomes which will be evaluated in this study affords the opportunity to apply explanatory models, such as *Quality of Medical Care* and the *Theoretical Risk Model for Mortality and Morbidity* by Donabedian (9), Krause et al. (43), and Krause et al. (44). No previous studies attempted studying 1-year mortality after TSCI in South Africa, let alone identifying factors influencing it. Using the Swedish health care system as standard may superimpose the validity of risk factors associated with mortality in South Africa. The assessment and monitoring of functioning will contribute to our understanding of the wider societal response to SCI.

Condition-specific action plans and policies are lacking in South Africa. This proposed study will provide an evidence-based foundation for sustaining life and improving functioning after TSCI through the development of clinical decision-making models that is based on injury-related and process-oriented factors. The hypothesis-driven approach applied in this study, combined with the population-based design, provides hospital management and policy makers with high quality evidence to assess the feasibility and cost-effectiveness of life-saving changes in processes/interventions in the future. Specifically related to the South African context, newly-injured patients are currently accepted to the only acute spinal cord injury unit based on need of surgery, without considering other contextual factors. The outcome of this study, i.e., development of a clinical decision-making model, could assist with the identification of those most at risk and ensure that they receive the time-sensitive care that is required. The data derived from this project will be published in peer reviewed, open access journals specifically interested in spinal cord injury management.

In conclusion, this study protocol offers a unique investigation of mortality and functioning (morbidity) after TSCI by determining the influence of acute care processes, as evident from acute clinical practice guidelines, on outcomes. This study will further identify the unmet health and functioning needs of persons with SCI in both context. This information could be used to adapt health care policies and practice guidelines.

## Author contributions

DC assisted with conceptualization of the project and secured funding for the project, re-drafted and provided input on the manuscript. AR assisted with fund procurement and reviewed the draft manuscript. NM, LN, and KW all assisted with study conceptualization and reviewing of the draft manuscript versions. CH assisted with drafting the ethical permission to conduct the study and reviewed the draft manuscript versions. CJ project leader, conceptualized the study design, secured funding for the project, draft the first full version of the manuscript, and addressed all co-authors' comments.

### Conflict of interest statement

The authors declare that the research was conducted in the absence of any commercial or financial relationships that could be construed as a potential conflict of interest.

## References

[1] Suarez NC Levi R Bullington J. Regaining health and wellbeing after traumatic spinal cord injury. J Rehabil Med. (2013) 45:1023–7. 10.2340/16501977-1226 24048205

[2] Dahlberg A Kotila M Kautiaien H Alaranta H. Functional independence in persons with spinal cord injury in Helsinki. J Rehabil Med. (2003) 35:217–20. 10.1080/16501970306092 14582553

[3] Joseph C Rhoda A Mji G Statham S Mlenzana N De Wet C Changes in activity limitations and predictors of functional outcome of patients with spinal cord injury following in-patient rehabilitation. S Afr J Physiother. (2013) 69:47–53. 10.4102/sajp.v69i1.371

[4] Carpenter C Forwell SJ Jongbloed LE Backman C. Community participation after spinal cord injury. Arch Phys Med Rehabil. (2007) 88:427–433. 10.1016/j.apmr.2006.12.043 17398242

[5] Barclay L Mcdonald R Lentin P. Social and community participation following spinal cord injury: a critical review. Int J Rehabil Res. (2015) 38:1–19. 10.1097/MRR.0000000000000085 25305008

[6] Consortium For Spinal Cord Medicine Early acute management in adults with spinal cord injury: a clinical practice guideline for health-care professionals. J Spinal Cord Med. (2008) 31:403–79. 10.1080/10790268.2008.11760744 18959359 PMC2582434

[7] Lalwani S Singh V Trikha V Sharma V Kumar S Bagla R. Mortality profile of patients with traumatic spinal injuries at a level I trauma care centre in India. Indian J Med Res. (2014) 140:40–45. 25222776 PMC4181158

[8] Chamberlain JD Meier S Mader L Von Groote PM Brinkhof MWG. Mortality and longevity after a spinal cord injury: systematic review and meta-analysis. Neuroepidemiology (2015) 44:182–98. 10.1159/000382079 25997873

[9] Donabedian A. The quality of medical care. Science (1978) 200:856–64. 10.1126/science.417400 417400

[10] Durlak JA Studying program implementation is not easy but it is essential. Prev Sci. (2015) 16:1123–1127. 10.1007/s11121-015-0606-3 26399607

[11] Saetren H Facts and myths about research on public policy implementation: out-of-fashion, allegedly dead, but still very much alive and relevant. Policy Stud J. (2005) 33:559–82. 10.1111/j.1541-0072.2005.00133.x

[12] Bernhard M Gries A Kremer P Bottiger BW. Spinal cord injury (SCI)–prehospital management. Resuscitation (2005) 66:127–39. 10.1016/j.resuscitation.2005.03.005 15950358

[13] Consortium for Spinal Cord Medicine Outcomes following traumatic spinal cord injury: clinical practice guidelines for health-care professionals. J Spinal Cord Med. (2000) 23:289–316. 10.1080/10790268.2000.11753539 17536300

[14] Jug M Kejzar N Vesel M Al Mawed S Dobravec M Herman S. Neurological Recovery after traumatic cervical spinal cord injury is superior if surgical decompression and instrumented fusion are performed within 8 hours versus 8 to 24 hours after injury: a single center experience. J Neurotrauma (2015) 32:1385–92. 10.1089/neu.2014.3767 25658291

[15] Divanoglou A Seiger A Levi R. Acute management of traumatic spinal cord injury in a Greek and a Swedish region: a prospective, population-based study. Spinal Cord (2010) 48:477–82. 10.1038/sc.2009.160 20029396

[16] Divanoglou A Westgren N Seiger Å Hulting C Levi R. Late mortality during the first year after acute traumatic spinal cord injury: a prospective, population-based study. J Spinal Cord Med. (2010) 33:117–27. 10.1080/10790268.2010.11689686 20486530 PMC2869273

[17] Parent S Barchi S Lebreton M Casha S Fehlings MG. The impact of specialized centers of care for spinal cord injury on length of stay, complications, and mortality: a systematic review of the literature. J Neurotrauma (2011) 28:1363–70. 10.1089/neu.2009.1151 21410318 PMC3143414

[18] Divanoglou A Westgren N Bjelak S Levi R. Medical conditions and outcomes at 1 year after acute traumatic spinal cord injury in a Greek and a Swedish region: a prospective, population-based study. Spinal Cord (2010) 48:470–6. 10.1038/sc.2009.147 20029392

[19] Bjerner B Astrom J. Treatment of paraplegic patients at the danderyd rehabilitation clinic. Scand J Rehabil Med. (1970) 2:27–8. 5523815

[20] Nordgren C Levi R Ljunggren G Seiger A. Societal services after traumatic spinal cord injury in Sweden. J Rehabil Med. (2003) 35:121–6. 10.1080/16501970310010466 12809194

[21] Joseph C Delcarme A Vlok I Wahman K Phillips J Nilsson Wikmar L. Incidence and aetiology of traumatic spinal cord injury in Cape Town, South Africa: a prospective, population-based study. Spinal Cord (2015) 53:692–6. 10.1038/sc.2015.51 25823800

[22] Sothmann J Stander J Kruger N Dunn R. Epidemiology of acute spinal cord injuries in the Groote Schuur Hospital Acute Spinal Cord Injury (GSH ASCI) Unit, Cape Town, South Africa, over the past 11 years. S Afr Med J. (2015) 105:835–839. 10.7196/SAMJnew.8072 26428588

[23] Fielingsdorf K Dunn RN. Cervical spine injury outcome–a review of 101 cases treated in a tertiary referral unit. S Afr Med J. (2007) 97:203–207. 17440669

[24] Joseph C Nilsson Wikmar L. Prevalence of secondary medical complications and risk factors for pressure ulcers after traumatic spinal cord injury during acute care in South Africa. Spinal Cord (2015) 54:535–9. 10.1038/sc.2015.189 26481710

[25] Ataguba JE Akazili J Mcintyre D. Socioeconomic-related health inequality in South Africa: evidence from General Household Surveys. Int J cEquity Health (2011) 10:48. 10.1186/1475-9276-10-48 22074349 PMC3229518

[26] Divanoglou A Levi R. Incidence of traumatic spinal cord injury in Thessaloniki, Greece and Stockholm, Sweden: a prospective population-based study. Spinal Cord (2009) 47:796–801. 10.1038/sc.2009.28 19350044

[27] Kirshblum SC Burns SP Biering-Sorensen F Donovan W Graves DE Jha A. International standards for neurological classification of spinal cord injury (revised 2011). J Spinal Cord Med. (2011) 34:535–46. 10.1179/204577211X13207446293695 22330108 PMC3232636

[28] Joseph C Andersson N Bjelak S Giesecke K Hultling C Nilsson Wikmar L. Incidence, aetiology and injury characteristics of traumatic spinal cord injury in Stockholm, Sweden: a prospective, population-based update. J Rehabil Med. (2017) 49:431–6. 10.2340/16501977-2224 28451696

[29] Devivo M Biering-Sorensen F Charlifue S Noonan V Post M Stripling T. International spinal cord injury core data set. Spinal Cord. (2006) 44:535–40. 10.1038/sj.sc.3101958 16955073

[30] Pressure ulcer prevention and treatment following spinal cord injury A clinical practice guideline for health-care professionals. J Spinal Cord Med. (2001) 24(Suppl. 1):S40–101. 10.1080/10790268.2001.11753592 11958176

[31] Kirchberger I Cieza A Biering-Sorensen F Baumberger M Charlifue S Post MW. ICF Core Sets for individuals with spinal cord injury in the early post-acute context. Spinal Cord (2010) 48:297–304. 10.1038/sc.2009.128 19786973

[32] Cieza A Kirchberger I Biering-Sorensen F Baumberger M Charlifue S Post MW. ICF Core Sets for individuals with spinal cord injury in the long-term context. Spinal Cord (2010) 48:305–12. 10.1038/sc.2009.183 20065984

[33] Kalpakjian CZ Scelza WM Forchheimer MB Toussaint LL. Preliminary reliability and validity of a spinal cord injury secondary conditions scale. J Spinal Cord Med. (2007) 30:131. 10.1080/10790268.2007.11753924 17591225 PMC2031942

[34] Waring WP Biering-Sorensen F Burns S Donovan W Graves D Jha A. 2009 Review and revisions of the international standards for the neurological classification of spinal cord injury. J Spinal Cord Med. (2010) 33:346–52. 10.1080/10790268.2010.11689712 21061894 PMC2964022

[35] Graves DE Frankiewicz RG Donovan WH. Construct validity and dimensional structure of the ASIA motor scale. J Spinal Cord Med. (2006) 29:39–45. 10.1080/10790268.2006.11753855 16572564 PMC1864793

[36] Almeida C Coelho JN Riberto M. Applicability, validation and reproducibility of the Spinal Cord Independence Measure version III (SCIM III) in patients with non-traumatic spinal cord lesions. Disabil Rehabil. (2016) 38:2229–34. 10.3109/09638288.2015.1129454 26800790

[37] Bluvshtein V Front L Itzkovich M Aidinoff E Gelernter I Hart J. SCIM III is reliable and valid in a separate analysis for traumatic spinal cord lesions. Spinal Cord (2011) 49:292–296. 10.1038/sc.2010.111 20820178

[38] Catz A Itzkovich M Agranov E Ring H Tamir A. SCIM-Spinal Cord Independence Measure: a new disability scale for patients with spinal cord lesions. Spinal Cord (1997) 35:850–6. 10.1038/sj.sc.3100504 9429264

[39] Magasi SR Heinemann AW Whiteneck GG Participation following traumatic spinal cord injury: an evidence-based review for research : report on the national institute on disability and rehabilitation research spinal cord injury measures meeting. J Spinal Cord Med. (2008) 31:145–56. 10.1080/10790268.2008.11760705 18581661 PMC2565477

[40] Dijkers MP. Quality of life of individuals with spinal cord injury: a review of conceptualization, measurement, and research findings. J Rehabil Res Dev. (2005) 42:87–110. 10.1682/JRRD.2004.08.0100 16195966

[41] International perspectives on spinal cord injury (2012). International perspectives on spinal cord injury. Geneva: International Perspectives on Spinal Cord Injury, World Health Organization.

[42] National Rehabilitation Policy (2001). National Rehabilitation Policy. Pretoria: Pretoria Republic of South Africa.

[43] Krause JS Saunders LL Dipiro ND Reed KS. theoretical risk and prevention model for secondary health conditions and mortality after SCI: 15 years of research. Top Spinal Cord Injury Rehabil. (2013) 19:15–24. 10.1310/sci1901-15 23459002 PMC3584350

[44] Krause JS Zhai Y Saunders LL Carter RE. Risk of mortality after spinal cord injury: an 8-year prospective study. Arch Phys Med Rehabil. (2009) 90:1708–15. 10.1016/j.apmr.2009.04.020 19801060 PMC3181069

