# Supplementary material for: Strengthening Health Systems for Persons With Traumatic Spinal Cord Injury in South Africa and Sweden: A Protocol for a Longitudinal Study of Processes and Outcomes
Source: Front Neurol. 2018 Jun 14;9:453. doi: 10.3389/fneur.2018.00453 (PMC6011127; doi:10.3389/fneur.2018.00453)
Supplement: Supplementary file 1 [file Table_1.docx]

| **Supplementary material 1.** Mapping of items in outcome measures to the ICF categories | | | |
| --- | --- | --- | --- |
| ***Outcomes*** | | ***Measure used*** | ***ICF category addressed*** |
| ***Health outcomes*** | |  |  |
| 1.Survival status | | *Alive or dead* |  |
| 2.Secondary complications  ***Functioning outcomes*** | | *Secondary health conditions scale-SCI* | *b810 (Protective functions of the skin)*  *b298 (Sensory functions and pain, other specified)*  *b735 (muscle tone functions)*  *b710 (mobility of joint functions)*  *b620 (urination functions)*  *b525 (defecation function)*  *b435 (immunological system functions)*  *b640 (sexual functions)*  *Autonomic dysreflexia (not in core set)*  *b440 (respiration functions)*  *b280 (sensation of pain).* |
| 3. Neurological Classification | | *International Standards of assessment* | *b280 (pain sensation)*  *b265 (touch function)*  *b730 (muscle power functions)* |
| 4. Functional status | | *SCIM III* | *D550 (eating);d560 (drinking)*  *d510 (washing oneself); d540 (Dressing) d520 (Caring for body parts)*  *b440 (Respiration functions)*  *b620 (urination functions)*  *b525 (Defecation function)*  *d530 (Toileting)*  *d410 (Changing basic body positions)*  *d445 (hand and arm use)*  *d420 (transferring oneself)*  *d440 (fine hand use)*  *d450 (Walking)*  *d465 (moving around using equipment); d455 (moving around)*  *d430 (lifting and carrying objects).* |
| 5.Activities and Participation  6. Health-related Quality of life | *CHART (16 participation codes, the rest activity)*  *European Quality of Life 5D* | | *d730 (Relating with strangers)*  *d740 (Formal relationships)*  *d750 (Informal social relationships)*  *d760 Family relationships*  *d770 (Intimate relationships)*  *d820 (School education)*  *d825 Vocational training)*  *d830 (Higher education)*  *d850 (Remunerative employment)*  *d855 (Non-remunerative employment)*  *d870 (Economic self-sufficiency)*  *d920 (Recreation and leisure)*  *(CHART used to develop the data set)* |
