# Supplementary material for: Strengthening Health Systems for Persons With Traumatic Spinal Cord Injury in South Africa and Sweden: A Protocol for a Longitudinal Study of Processes and Outcomes
Source: Front Neurol. 2018 Jun 14;9:453. doi: 10.3389/fneur.2018.00453 (PMC6011127; doi:10.3389/fneur.2018.00453)
Supplement: Supplementary file 2 [file Table_2.docx]

| **Supplementary material 2. Definitions of secondary complications** | |
| --- | --- |
| **Secondary complication:** | **Definition** |
| *Pressure sore(s)* | These develop as a skin rash or redness and progress to an infected sore. Also called skin ulcers, bedsores and decubitus ulcers. |
| *Injury caused by loss of sensation* | Injury may occur because of a lack of sensation, such as burns from carrying hot liquids in the lap or sitting too close to a heater or fire. |
| *Muscle spasms (spasticity)* | Spasticity refers to uncontrolled, jerky muscle movements, such as uncontrolled muscle twitch or spasm. Often spasticity increases with infection or some kind of restriction, like a tight shoe or belt. |
| *Contractures* | A contracture is a limitation in range of motion caused by a shortening of the soft tissue around a joint, such as an elbow or hip. This occurs when a joint cannot move frequently enough through its range of motion. Pain often accompanies this problem. |
| *Heterotopic bone ossification* | This is an overgrowth of bone, often occurring after a fracture. Early signs include a loss of range of motion, local swelling and warmth at the area to the touch. This condition must be diagnosed by a physician. |
| *Diabetes mellit*us | Diabetes is a problem resulting from irregularities in blood sugar levels. Symptoms include frequent urination and excessive thirst. This condition is diagnosed by a physician. |
| *Bladder dysfunction* | Incontinent, bladder or kidney stones, kidney problems, urine leakage and urine back up are all symptoms of bladder dysfunction. NOTE: there is a separate item for urinary tract infections. |
| *Bowel dysfunction* | Diarrhea, constipation, “accidents”, and associated problems are signs of bowel dysfunction |
| *Urinary tract infections* | This includes infections such as cystitis and pseudomonas. Symptoms include pain when urinating, a burning sensation throughout the body, blood in the urine and cloudy urine. |
| *Sexual dysfunction* | This includes dissatisfaction with sexual functioning. Causes for dissatisfaction can be decreased sensation, changes in body image, difficulty in movement, and problems with bowel or bladder, like infections. |
| *Autonomic dysreflexia* | Autonomic dysreflexia, sometimes called hyperreflexia, results from interference in the body’s temperature regulating systems. Symptoms of dysreflexia include sudden rises in blood pressure and sweating, skin blotches, goose bumps, pupil dilation and headache. It can also as the body’s response to pain where an individual doesn’t experience sensation. |
| *Postural hypotension* | This involves a strong sensation of lightheadedness following a change in position. It is caused by a sudden drop in blood pressure. |
| *Circulatory problems* | Circulatory problems involve the swelling of veins, feet or the occurrence of blood clots. |
| *Respiratory problems* | Symptoms of respiratory infections or problems include difficulty in breathing and increased secretions. |
| *Chronic pain* | This is usually experienced as chronic tingling, burning or dull aches. It may occur in an area that has little to no feeling. |
| *Joint and muscle pain* | This includes pain in specific muscle groups or joints. People who must overuse a particular muscle group, such as shoulder muscles, or who put too much strain on their joints are at risk of developing pain. |
